# Tensile strength analysis of additively manufactured CM 247LC alloy specimen by employing machine learning classifiers

**DOI:** 10.1371/journal.pone.0305744

**Published:** 2024-07-29

**Authors:** Vijaykumar S. Jatti, Dhruv A. Sawant, Nitin K. Khedkar, Vinaykumar S. Jatti, Sachin Salunkhe, Marek Pagáč, Emad S. Abouel Nasr

**Affiliations:** 1 Symbiosis Institute of Technology, Symbiosis International (Deemed University), Pune, India; 2 Department of Biosciences, Saveetha School of Engineering, Saveetha Institute of Medical and Technical Sciences, Chennai, India; 3 Department of Mechanical Engineering, Gazi University Faculty of Engineering, Ankara, Türkiye; 4 Department of Machining, Assembly and Engineering Technology, Faculty of Mechanical Engineering, Ostrava-Poruba, Czechia; 5 Department of Industrial Engineering, College of Engineering, King Saud University, Riyadh, Saudi Arabia; G H Raisoni College of Engineering and Management, Pune, INDIA

## Abstract

Using a cutting-edge net-shape manufacturing technique called Additive Layer Manufacturing (ALM), highly complex components that are not achievable with conventional wrought and cast methods can be produced. As a result, the aerospace sector is paying closer attention to using this technology to fabricate superalloys based on nickel to develop the holistic gas turbine. Because of this, there is an increasing need for the mechanical characterisation of such material. Conventional mechanical testing is hampered by the limited availability of material that has been processed, especially given the large number of process factors that need to be assessed. Thus, the present study focuses on manufacturing CM247LC Ni-based superalloy with exceptional mechanical characteristics by laser powder bed fusion (L-PBF). This study evaluates the effect of input process variables such as laser power, scan speed, hatch distance and volumetric energy density on the mechanical performance of the LPBF CM247LC superalloy. The maximum value of as-built tensile strength obtained in the study is 997.81 MPa. Plotting Pearson’s heatmap and the Feature importance (F-test) was used in the data analysis to examine the impact of input parameters on tensile strength. The accuracy of the tensile strength data classification by machine learning algorithms, such as k-nearest neighbours, Naïve Baiyes, Support vector machine, XGBoost, AdaBoost, Decision tree, Random forest, and logistic regression algorithms, was 92.5%, 83.75%, 83%, 85%, 87.5%, 90%, 91.25%, and 77.5%, respectively.

## 1. Introduction

Many issues frequently encountered with traditional joining procedures can be resolved with laser welding, a high-power density yet low energy-input approach. Its main features are precise weld bead placement, quick heating and cooling, minimal distortion, flexible processing, and chances for product redesign [1]. Rapid prototyping, different from forming and material removal manufacturing procedures, refers to methods that create shaped objects by gradually creating or adding solid material [2]. The tiny Punch Creep (SPC) test method uses tiny amounts of material for each test. It allows for discrete location sampling, a successful instrument for ranking the raised temperature performance of alloys processed using ALM. This study will employ the SPC test to evaluate the impact of many critical process variables on the mechanical performance of the Ni-based Laser Powder Bed Fused (LPBF) superalloy CM247LC [3]. Technological developments in nickel-based superalloys and additive manufacturing techniques can lead to creative turbine designs. Selective laser melting (SLM) and selective electron beam melting (SEBM) of nickel-based superalloys offer clear benefits in this area. Additionally, nickel alloy component reclamation and repair can be accomplished using direct energy deposition (DED) methods [4]. The application of additive manufacturing (AM) in producing nickel-based alloys has witnessed significant adoption in both scholarly and industrial settings. On the other hand, AM processing of conventional Ni-based superalloys has been plagued by many cracking problems. Solidification, solid-state, and liquidation processes are the main types of cracking [5]. To create a shaped CM247LC black, the research on CM247LC employed the conventional method of Near-Netshape Hot Isostatic Pressing (NNSHIP) with sacrificial low carbon steel tooling that was constructed using Selective Laser Melting (SLM). The microstructure evaluation concentrated on the interior microstructure and the external components to ascertain the depth of the Fe-diffusion layer [6]. The primary motivation behind layer manufacturing approaches, such as rapid prototyping (RP), shifted from producing prototypes to quick tooling (RT) and quick manufacturing (RM) [7]. Producing complex-shaped functional metallic components, such as metals, alloys, and metal matrix composites (MMCs), is the current emphasis of additive manufacturing (AM) development in order to meet the demanding needs of the aerospace, defence, automotive, and biomedical industries [8]. The gamma prime volume fraction appeared to be connected with the ranking of the superalloys based on the degree of cracking encountered (CMSX-4 exhibiting the greatest severity, followed by CM247LC DS and IN6203DS, which exhibited no indication of cracking at all) [9]. Observations using a transmission electron microscope on the slightly distorted specimens show that, in contrast to earlier studies, tightly coupled dislocations cutting through γ′ precipitates govern all specimens’ plastic deformation. The relationship between the yield strength and precipitate size is explored in light of these experimental findings [10]. The CM247LC superalloy’s Type-I hot corrosion behaviour is assessed at 950°C in the air against low (3 to 4), intermediate (7 to 9), and high (12 to 14 mg cm−2) Na2SO4 deposits. The temperature exposure ranges from five minutes to a thousand hours [11]. When the deformation temperature is lower than 800°C, it is discovered that the alloy’s yield strength only varies within a narrow range with temperature.

Conversely, the strength drastically drops as the temperature rises [12]. This work aims to estimate the density of cracks in the γ′-strengthened Ni superalloy CM247LC by developing a melt pool temperature model by laser powder bed fusion (LPBF) [13]. This study examines the viability of using MIM to manufacture parts from the nickel-based superalloy CM247LC. CM247LC presents a significant obstacle to MIM processing. The strength potential is very high because of the high aluminium content; however, the sintering capacity is severely limited [14]. Analysis is done on the impact of process parameters on the microstructure, porosity, and formation of cracks in the nickel-based alloy CM247LC, which is produced additively. The viability of producing low-porosity, crack-free CM247LC samples via direct laser deposition (DLD) is investigated [15]. MAD542, an experimental printable superalloy based on nickel and enhanced by γ′, is suggested. Laser powder bed fusion (LPBF) produced a crack-free component with less than 0.06% defect by optimising the procedure [16]. Authors in [17], discovered that the energy per layer was a crucial component in printing fully dense AlSi12 samples through the SLM technique. The variable energy per layer and printing area are correlated with the anisotropy of the SLM-built samples. Optimal ranges of energy per layer were used to print fully dense SLM-built AlSi12 samples. Authors in [18] examined how two distinct scanning methods affected the creation of 17-4PH stainless steel samples. These samples were created utilizing the corresponding techniques, and they were evaluated both before and after heat treatment. Relative density, microstructural phase composition, and microhardness were the main subjects of analysis. The samples printed using the double scan method had a higher relative density than samples printed using the single scan strategy, according to the results. Furthermore, the increased martensite phase distribution over preserved austenite in the double scan samples was responsible for their higher hardness. Moreover, the original samples’ heat treatment produced a homogeneous distribution of the tempered martensite-dominant phase with little austenite retained, improving their hardness to a level similar to that of wrought samples that had also undergone heat treatment. Table 1 depicts the Machine learning algorithms used in LPBF processes.

**Table 1:** Machine learning algorithms used in metal additive manufacturing processes.

Ref	Machine Learning Algorithm	AM Process
[19]	SVR, KNN	LPBF
[20]	SVR, KNN, SVM	LPBF
[21]	CNN	LPBF
[22]	SVR	LPBF
[23]	SVM, CNN	LPBF
[24]	FFNN, CNN	LPBF
[25]	CNN	LPBF
[26]	Backpropagation neural network	LPBF
[27]	Deep learning-based neural network	LPBF
[28]	Spectral convolutional neural networks	SLM

**Note:** SVM: Support vector machine, ANN: Artifcial neural network, ML: Machine learning, SVR: Support vector regression, KNN: K-nearest neighbor, LPBF: Laser powder bed fusion, SLM: Selective laser melting, CNN: Convolutional neural network, FFNN: Feed forward neural network

Aero-engine parts require the best tensile strength estimation of AM CM 247LC alloy components. Even though machine learning has shown promise in predictive modelling, assessing its effectiveness and selecting the optimal method for this specific use takes time and effort. It is still required. Moreover, there needs to be more study in this field and a lack of comprehensive comparison and analysis of different algorithms. This research study aims to compare the predictive power of machine learning algorithms, specifically K-nearest Neighbor, Naïve Bayes, Support Vector Machine, XGBoost, AdaBoost, Decision Tree, Random Forest, and Logistic Regression, in predicting the exact tensile strength of Metal Additively Manufactured CM 247LC alloy.

## 2. Materials and methods

**Fig 1:**
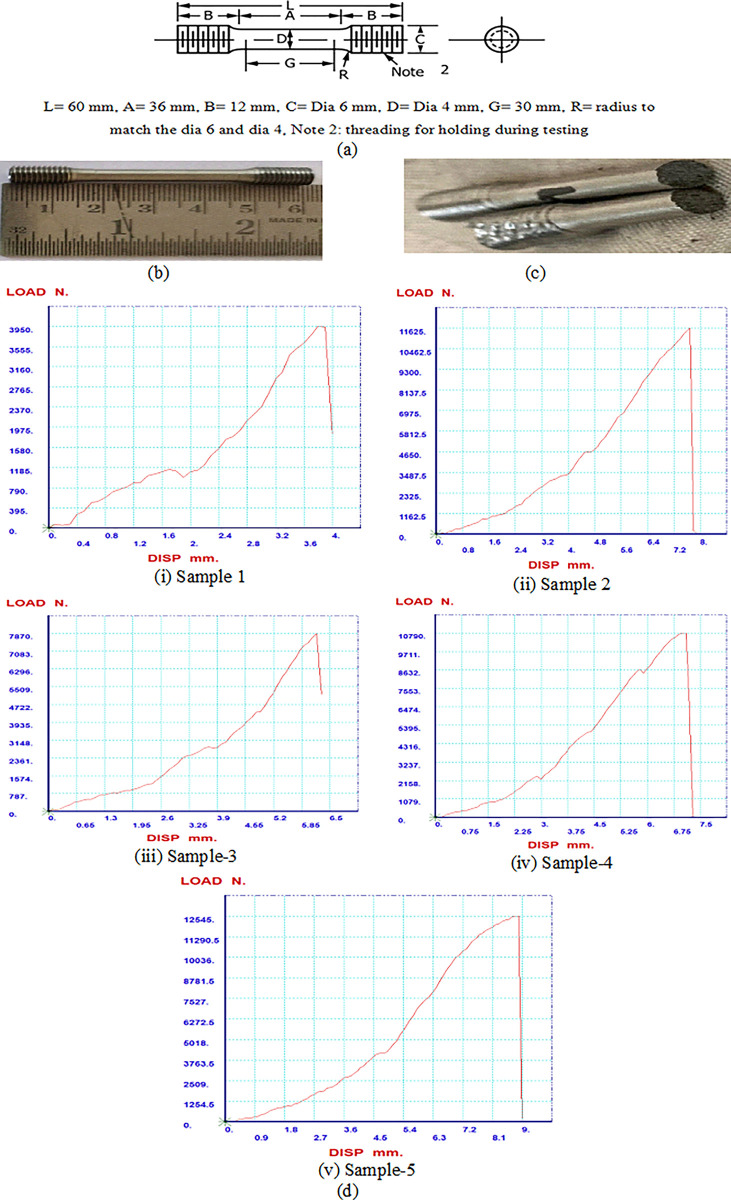
CM 247 specimens. (a) Specimen drawing as per ASTM E8 [29], (b) CM247 printed specimen (c) CM247 specimen after failure (d) Load versus displacement graph.

CM 247LC powder was chosen as the feedstock material for the study because it’s employed in the fabrication of Gas Generator-Nozzle Guide Vane, Jet Fuel Starter and Aerofoils. Sandvik Osprey Ltd. provided the atomized argon gas that was used to make the metal powder for this project, refer Table 2 for its chemical composition. Specimens were fabricated using the Renishaw model RenAM500. The specimens underwent post-processing after printing, which included wire EDM, bead blasting, and support removal. Tensile specimens were modelled (ref Fig 1A) and printed as per ASTM E8 standard (refer Fig 1B) [29] and specimens were tested using Computer Control Electro Hydraulic Type universal testing machine of make- TE Forcespeed Co. and model- WAW-1000E. Strain rate/crosshead velocity during tensile testing used is 5 mm/min. Fig 1C depicts the specimen after tensile test.

**Table 2:** Chemical composition of CM 247LC powder.

Element	W	Co	Cr	Al	Ta	Hf	Ti
wt %	9.32	8.71	8.26	5.54	3.12	1.21	0.73
Element	Mo	Si	Fe	C	Nb	O	Ni
wt%	0.54	0.12	0.08	0.08	0.05	<100 ppm	Bal

Table 3 shows the volumetric energy density sample readings for corresponding trial conduction. The volumetric energy density method indicates the bulk energy input to the process. The volumetric energy density is calculated as per Eq 1 [30]:

$$VED=\frac{P}{\nu \times h\times t}$$ (1)

**Table 3:** Experimental layout and corresponding volumetric energy density.

Sample No	Laser Power (W)	Scan Speed (mm/s)	Hatch Spacing (mm)	Layer Thickness (mm)	Volumetric Energy Density (VED) (J/mm^3^)	Tensile Strength MPa
1	220	800	0.1	0.04	68.75	314.157
2	235	850	0.11		62.83	924.541
3	250	900	0.12		57.87	625.975
4	250	900	0.12		57.87	858.280
5	235	850	0.11		62.83	997.818

VED is volumetric energy density in J/mm^3^, P is laser power in W, ν is scan speed in mm/s, h is hatch spacing in mm, and t is layer thickness in mm. Post processing included wire EDM, support removal, bead blasting, hot isostatic pressing (HIP) followed by solution heat treatment (ST) and ageing heat treatment. Hot Isostatic Pressing: 1250°C/2 h and 140 MPa, Solution heat-treatment: 1260°C/2 h and Ageing heat treatment: 870°C/16 h. To classify and predict the best label for tensile strength, different machine learning classification algorithms such as K-nearest Neighbor (kNN classification), Naïve Bayes Classification (NB), Support Vector Machine classification (SVM or SVC), XGBoost (eXtreme Gradient Boosting), AdaBoost (Adaptive Boosting), Decision Tree, Random Forest and Logistic Regression Classification were applied on the dataset. Data was synthetically generated using synthetic data generation for more accurate label prediction and to improve data quality. Synthetic data generation is creating new data automatically using computer simulations or algorithms or manually using tools like Excel to replace real-world data. Synthetic data has several significant advantages, including reducing restrictions on using sensitive or regulated data, modifying data to fit circumstances that accurate data cannot, and creating sizable training datasets without the need for manual data labelling. Table 2 shows the labelled class for tensile strength classification as 1 & 0 by considering the average tensile strength values as 744.13 MPa. Training and testing ratio considered for present study is 80:20 of the dataset.

Fig 1D depicts the load versus displacement curves for sample-1 to 5.

### 2.1 K-Nearest Neighbor classification

**Fig 2:**
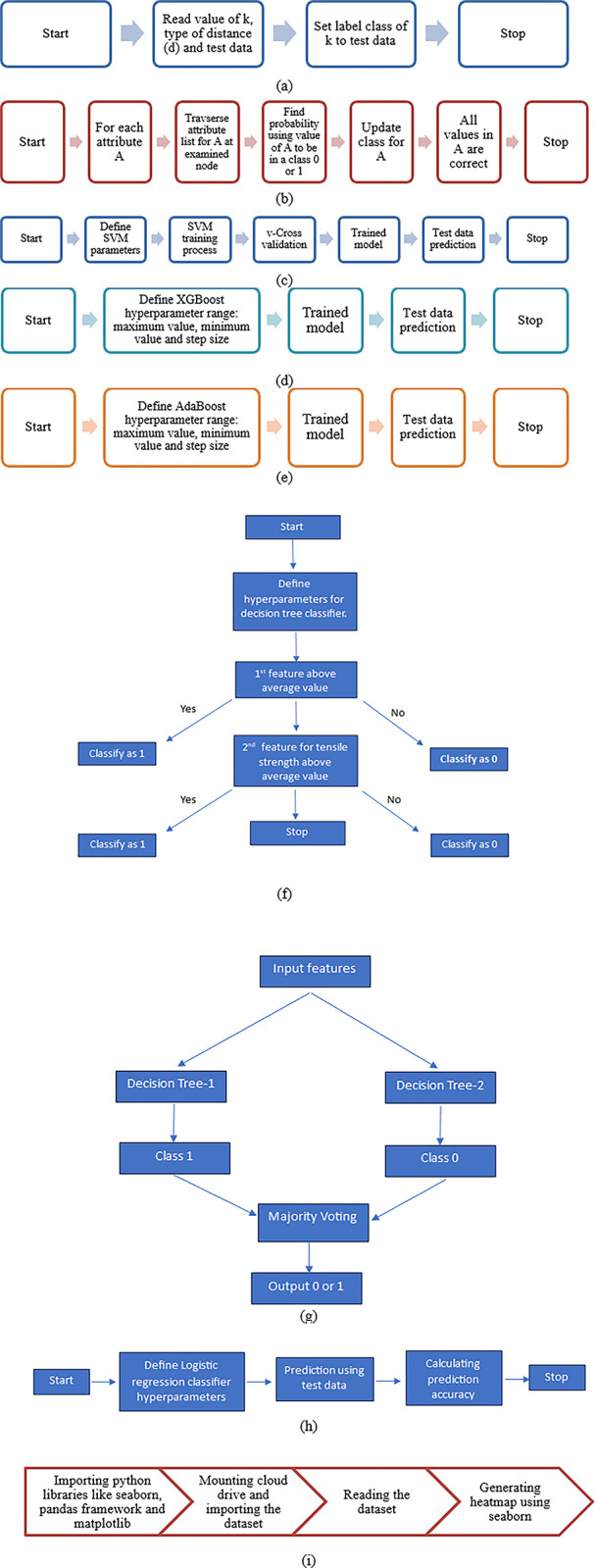
Machine learning algorithms. (a) kNN Classification Algorithm workflow (b) Naïve Bayes classification algorithm workflow (c) SVM classification algorithm workflow (d) XGBoost algorithm workflow (e) AdaBoost algorithm workflow (f) Decision Tree classification algorithm workflow (g) Random Forest classification workflow (h) Logistic Regression classifier workflow (i) Pearson’s heatmap workflow.

K-nearest neighbours are one supervised learning technique applied to regression and classification. KNN aims to predict the appropriate class for the test data by calculating the distance between the test data and the training points [31]. The K spots closest to the test data should then be selected. When the KNN algorithm calculates the probability that the test data will belong to each training data class for "K," the class with the highest probability will be selected. In the regression scenario, the value is the mean of the selected "K" training points. The KNN classification algorithm’s workflow for the current investigation is shown in Fig 2A.

### 2.2 Naïve Bayes Classification

Naive bayes is a simple classifier construction technique. Referring to Fig 2B, these models represent issue situations using feature vectors and give class labels to them based on a finite set of class labels. A family of methods with a common principle—that is, given the class variable, all naive Bayes classifiers assume that the value of a particular feature is independent of the value of any other feature—is used to train these classifiers instead of a single methodology [31].

### 2.3 Support Vector Machine classification

Support Vector Machine (SVM), a supervised machine learning technique, is used for regression and classification. Nevertheless, regression problems are most suited for application in classification issues. Finding the optimal hyperplane in an N-dimensional space to partition data points into different feature space classes is the main objective of the SVM method. The hyperplane aims to keep as big a buffer as possible between the closest points of different classes. The number of features determines the hyperplane’s dimension; see Fig 2C. When input features are limited to two, the hyperplane can be considered a line. The hyperplane changes into a 2-D plane if there are three input features [31].

### 2.4 XGBoost (eXtreme Gradient Boosting)

XGBoost is a distributed gradient boosting toolkit designed for machine learning model training that prioritises efficiency and scalability. It is an ensemble learning method that combines the predictions of multiple weak models to get a more robust prediction. Due to its ability to handle enormous datasets and attain state-of-the-art performance in various machine learning tasks, such as regression and classification, Extreme Gradient Boosting, or XGBoost, is a machine learning technique that has acquired popularity and broad implementation [31]. The workflow of the XGBoost classification algorithm for the current investigation is shown in Fig 2D.

### 2.5 AdaBoost (Adaptive Boosting)

Ada-boost, or Adaptive Boosting, is one ensemble boosting classifier that Yoav Freund and Robert Schapire proposed in 1996. It integrates many classifiers to increase classifier accuracy. An approach for iterative ensembles is called AdaBoost. The AdaBoost classifier combines many underperforming classifiers to produce a robust and accurate classifier. The core principle of Adaboost is to ensure accurate predictions of anomalous events by training the data sample and modifying the classifier weights in each iteration [31]. The workflow of the AdaBoost classification algorithm for the current investigation is shown in Fig 2E.

### 2.6 Decision tree

As shown in Fig 2F, a decision tree is a tree structure in which the leaf nodes indicate the result of the algorithm, the branches represent rules, and the core nodes represent features. Decision trees are similar to flowcharts. It is a versatile supervised machine-learning technique that may be used for classification and regression problems. It is one of the most robust algorithms. It is one of the most powerful machine learning algorithms since Random Forest trains on different subsets of training data. [31]

### 2.7 Random forest

In a random forest classification, different decision trees are produced using distinct random subsets of the data and characteristics; see Fig 2G. As an expert, every decision tree guides how to classify the data. Predictions are created by calculating the result of each decision tree and choosing the most often accepted response [31].

### 2.8 Logistic Regression Classification

In logistic regression, a supervised machine learning algorithm, a logistic function, also known as a sigmoid function, is used to produce a probability value between 0 and 1 based on inputs that are independent variables (see Fig 2H). For instance, Class 0 and Class 1 are the two classes. If the logistic function value of an input exceeds the threshold value of 0.5, it is classed as Class 1 or Class 0. It is called regression because it is essentially used for classification problems and is a continuation of linear regression [31].

Furthermore, the AUC-ROC curve was used to analyse the feature importance of each input parameter on the tensile strength. AUC-ROC analysis is a useful tool for assessing binary classification models’ performance and determining which ones are useful for a given task. Plot the ROC curve, with TPR on the y-axis and FPR on the x-axis. Each point on the curve represents a different threshold setting. Calculate the area under the ROC curve. A perfect classifier would have an AUC of 1. Compare the AUC values of different classifiers or different models to determine which one performs better. Different feature importance plots like ANOVA and Pearson’s heatmap were plotted. The acronym for analysis of variance is ANOVA. It is a statistical test that examines the statistical distinctions between the data’s numerical and categorical feature sets. Generally speaking, it looks for relationship patterns between the different data features. Analysing the variances of the samples taken from a population may also be used to test hypotheses by determining if two or more population means are equal. A statistical indicator of the linear relationship between two variables is the correlation coefficient, sometimes the Pearson correlation coefficient. The correlation coefficient is another name for the Pearson correlation. The application of the Pearson correlation coefficient spans numerous disciplines, including biology, sociology, and psychology. The Pearson correlation coefficient is frequently used in psychology to assess the extent to which two constructs—intelligence and academic achievement—are related. The Pearson correlation coefficient can be used in sociological studies to investigate the relationship between education level and income. Refer to Fig 2I, which depicts a person’s heatmap workflow.

## 3. Results and discussions

This section depicts the tensile strength results together with a machine learning technique in order to estimate the tensile strength. Table 4 displays the experimental settings employed in the research, and the matching observed tensile strength values.

**Table 4:** Observed values along with experimental design layout.

Sr. No.	Laser Power (W)	Scan Speed (mm/s)	Hatch Spacing (mm)	Volumetric Energy	Tensile Strength (MPa)	Labelled classes
				Density (J/mm^3^)		
1	235.05	850.18	0.12	62.92	924.57	1
2	249.96	900.03	0.48	58.15	625.84	0
3	250.07	899.99	0.19	57.85	858.26	1
4	…	…	…	…	…	
…	…	…	…	…	…	
100	220.0398	800.0884	0.118026	68.80509	314.2183	0

The laser power determines the intensity of the laser beam used to melt the metal powder. Increased laser power means more energy input and possibly more thorough powder melting. Increasing the laser’s power may improve the powder particles’ fusing, increasing the tensile strength. There is an ideal range, though; using too much power can lead to problems, including overheating and diminished mechanical properties. In the present study, 235 W resulted in the best tensile strength. The rate at which the laser scans the powder bed is called the scan speed. Quicker scan times cut down on how much time the laser spends in each location. More energy may be transferred to the material at lower scan rates, resulting in better fusion and increased tensile strength. On the other hand, abnormally slow speeds could lead to overheating and other problems. Achieving the ideal balance is essential. In the present study, 850 mm/sec resulted in the best tensile strength. The vertical distance between consecutive laser scan pathways is called the hatch distance, sometimes called layer thickness. It affects the thickness of the layers and, as a result, the general geometry of the printed part. In the present study, a hatch distance of 0.11 mm resulted in the best tensile strength.

When using laser powder bed fusion (LPBF) techniques, such as the additive production of CM 247LC specimens, volumetric energy density is essential. The quantity of energy imparted to a unit volume of material during manufacturing is known as the volumetric energy density. It is computed considering hatch distance, scan speed, and laser power. The microstructure and mechanical qualities of the finished product are impacted by the volumetric energy density, which also affects the extent of melting and consolidation of the metal powder. There is usually an ideal range of volumetric energy density for the optimum tensile strength. Below this point, adequate energy could prevent the powder particles from fusing poorly, decreasing tensile strength. Excessive energy above this threshold might result in problems like overheating, residual strains, and diminished mechanical characteristics. In the present study, the volumetric energy density of 62.83 J/mm^3^ resulted in the best tensile strength.

**Fig 3:**
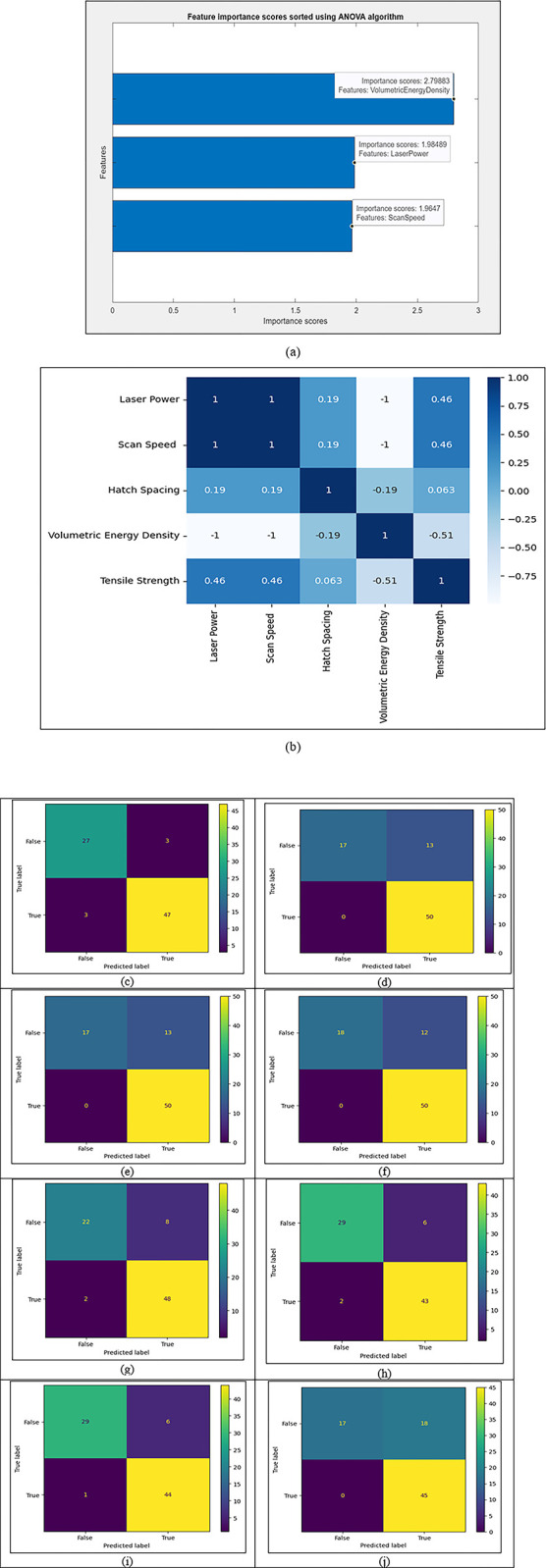
Machine learning results. (a) ANOVA feature importance plot (b) Pearson’s heatmap (c) Confusion matrix for kNN (d) Confusion matrix for Naïve Bayes (e) Confusion matrix for SVM (f) Confusion matrix for XGBoost (g) Confusion matrix for ADABoost (h) Confusion matrix for decision tree (i) Confusion matrix for random forest (j) Confusion matrix for logistic regression classifier.

ANOVA, also known as analysis of variance, is used to calculate the difference between the significance of 2 groups or features. In the Fig 3A ANOVA feature importance plot, the scan speed has the most minor difference, around 1.96, with laser power being 1.98 and Volumetric energy density being 2.79 are the most significant parameter.

Fig 3B above shows the heatmap analysis for each feature’s significance on the tensile strength value. The input parameters or the independent values considered are volumetric energy density, scan speed, hatch spacing and laser power. The dependent value or the output value is the tensile strength. It can be analysed that volumetric energy density (-0.51) laser power and scan speed are the most significance on the tensile strength, giving a value of 0.46. In contrast, hatch spacing is 0.063.

### Usually, the confusion matrix has four components

- The quantity of occurrences that the model accurately predicts to be positive—that is, positive instances—is known as True Positives (TP).
- The number of occurrences that the model mistakenly predicts as positive (i.e., forecasts a positive outcome when it’s actually negative) is known as False Positives (FP).
- True Negatives (TN): The quantity of cases that the model accurately predicts to be negative, or that is, as negative examples.
- False Negatives (FN): The quantity of cases that the model forecasts as negative when it should have predicted a positive outcome, or vice versa.

With TPR on the y-axis and FPR on the x-axis, a ROC curve is shown.

### In a scientific context, the results of the confusion matrix suggest

Accuracy: Accuracy is the ratio of (TP + TN) / (TP + TN + FP + FN) and indicates how accurate the model’s predictions are overall. It shows the percentage of cases that were accurately classified out of all instances. Better overall performance is suggested by higher accuracy.

Precision is defined as TP / (TP + FP) and is the percentage of true positives among all cases that are projected to be positive. The precision of a model indicates its capacity to prevent false positives. Precision is essential in scientific research since false positives can result in costly errors or incorrect findings.

Recall (Sensitivity): Recall, sometimes referred to as sensitivity or true positive rate (TPR), is computed as TP / (TP + FN) and is the percentage of true positives that the model properly recognized. When the cost of false negatives is large, as in the detection of medical conditions, recall becomes crucial since missing a positive case can have dire repercussions.

Specificity: Defined as TN / (TN + FP), specificity is the percentage of true negatives that the model properly identified. When the cost of false positives is large, like in security or fraud detection, specificity is crucial since misclassifying a benign event as harmful can result in needless actions.

F1 Score: The F1 score offers a balance between recall and precision by taking the harmonic mean of the two criteria. 2 * (Precision * Recall) / (Precision + Recall) is the formula used to compute it. When there is an unequal distribution of classes or when it is necessary to reduce both false positives and false negatives, the F1 score can be helpful.

### 3.1 kNN classification

The confusion matrix shown in Fig 3C depicts the confusion matrix prediction summary of the correct and incorrect labels. The number of true positives is the value that is correctly predicted tensile strength value above the average value of tensile strength, which is 47 in this case. The number of true negatives is the value that is correctly predicted, the tensile strength value below the average value of the tensile strength, which, in this case, is 27. The accuracy of the kNN classification algorithm was calculated to be 92.5%. The classification report, which includes metrics like precision, recall, accuracy, and f1-score, is given in Table 5.

**Table 5:** Classification report kNN.

Class	Precision	Recall	F1-score	Support
0	0.90	0.90	0.90	30
1	0.94	0.94	0.94	50
Accuracy	-	-	0.93	80
Macro avg	0.92	0.92	0.92	80
Weighted avg	0.93	0.93	0.93	80

### 3.2 Naïve Bayes classification

The confusion matrix prediction summary of the correct and incorrect labels is displayed in Fig 3D confusion matrix. In this case, the value accurately predicted to have tensile strength above the average value of 50 is known as the number of true positives. In this case, the value that accurately predicts a tensile strength value below the average tensile strength value of 17 is the number of true negatives. It was determined that the Naïve Baiyes classification method has an accuracy of 83.75%. Table 6 provides the classification report containing parameters like f1-score, recall, accuracy, and precision.

**Table 6:** Classification report for Naïve Bayes.

Class	Precision	Recall	F1-score	Support
0	1.00	0.57	0.72	30
1	0.79	1.00	0.88	50
Accuracy	-	-	0.84	80
Macro avg	0.90	0.78	0.80	80
Weighted avg	0.87	0.84	0.82	80

### 3.3 Support Vector Machine classification

The confusion matrix in Fig 3E shows the prediction summary of the correct and wrong labels. The number of true positives in this scenario is the value precisely predicted to have tensile strength above the average value of 50. The number of true negatives is defined as the value that, in this case, is 17 and accurately forecasts a tensile strength value below the average tensile strength value. The accuracy of the support vector classification approach was found to be 83%. The classification report is shown in Table 7 and includes parameters such as recall, accuracy, precision, and f1-score.

**Table 7:** Classification report for SVM.

Class	Precision	Recall	F1-score	Support
0	1.00	0.57	0.72	30
1	0.79	1.00	0.88	50
Accuracy	-	-	0.84	80
Macro avg	0.90	0.78	0.80	80
Weighted avg	0.87	0.84	0.82	80

### 3.4 eXtreme Gradient Boosting

The prediction summary of the correct and incorrect labels is displayed in Fig 3F confusion matrix. In this case, the number of true positives is the value that is precisely predicted to have tensile strength above the average value of 50. In this instance, the value that is 18 and correctly predicts a tensile strength value below the average tensile strength value is known as the number of true negatives. It was discovered that the XGBoost classification method has an 85% accuracy rate. Table 8 displays the categorisation report and metrics like recall, accuracy, precision, and f1-score.

**Table 8:** Classification report for XGBoost.

Class	Precision	Recall	F1-score	Support
0	1.00	0.57	0.72	30
1	0.79	1.00	0.88	50
Accuracy	-	-	0.84	80
Macro avg	0.90	0.78	0.80	80
Weighted avg	0.87	0.84	0.82	80

### 3.5 Adaptive boosting

The prediction summary of the correct and incorrect labels is displayed in Fig 3G confusion matrix. The number precisely predicted to have tensile strength above the average value of 48 is the number of true positives in this situation. In this instance, the value of 22 correctly predicts a tensile strength value below the average tensile strength value, which is known as the number of true negatives. The adaptive boosting classification method was discovered to have an 87.5% accuracy rate. Table 9 displays the categorisation report and metrics like recall, accuracy, precision, and f1-score.

**Table 9:** Classification report for AdaBoost.

Class	Precision	Recall	F1-score	Support
0	1.00	0.57	0.72	30
1	0.79	1.00	0.88	50
Accuracy	-	-	0.84	80
Macro avg	0.90	0.78	0.80	80
Weighted avg	0.87	0.84	0.82	80

### 3.6 Decision tree classification

The prediction summary of the correct and incorrect labels is displayed in Fig 3H confusion matrix. The number predicted to have tensile strength above the average value of 43 is the number of true positives in this situation. In this instance, the value that is 29 and correctly predicts a tensile strength value below the average tensile strength value is known as the number of true negatives. The decision tree classification method was discovered to have a 90% accuracy rate. Table 10 displays the categorisation report and metrics like recall, accuracy, precision, and f1-score.

**Table 10:** Classification report for decision tree.

Class	Precision	Recall	F1-score	Support
0	0.94	0.83	0.88	35
1	0.88	0.96	0.91	45
Accuracy	-	-	0.90	80
Macro avg	0.91	0.89	0.90	80
Weighted avg	0.90	0.90	0.90	80

### 3.7 Random forest classification

The confusion matrix in Fig 3I shows the prediction summary of the right and wrong labels. The number of true positives, in this case, is the number that is precisely predicted to have tensile strength above the average value of 44. The number of true negatives is the value that, in this case, is 29 and accurately forecasts a tensile strength value below the average tensile strength value. The accuracy rate of the decision tree classification approach was found to be 91%. The classification report and metrics, including recall, accuracy, precision, and f1-score, are shown in Table 11.

**Table 11:** Classification report for random forest.

Class	Precision	Recall	F1-score	Support
0	0.97	0.83	0.89	35
1	0.88	0.98	0.93	45
Accuracy	-	-	0.91	80
Macro avg	0.92	0.90	0.91	80
Weighted avg	0.92	0.91	0.91	80

### 3.8 Logistic regression classifier

The confusion matrix in Fig 3J shows the prediction summary of the right and wrong labels. The number of true positives, in this case, is the number that is precisely predicted to have tensile strength above the average value of 45. The number of true negatives is the value that, in this case, is 17 and accurately forecasts a tensile strength value below the average tensile strength value. It was shown that the accuracy rate of the logistic regression classifier approach is 78%. The classification report and metrics, including recall, accuracy, precision, and f1-score, are shown in Table 12.

**Table 12:** Classification report for logistic regression classifier.

Class	Precision	Recall	F1-score	Support
0	1.00	0.49	0.65	35
1	0.71	1.00	0.83	45
Accuracy	-	-	0.78	80
Macro avg	0.86	0.74	0.74	80
Weighted avg	0.84	0.78	0.75	80

**Fig 4:**
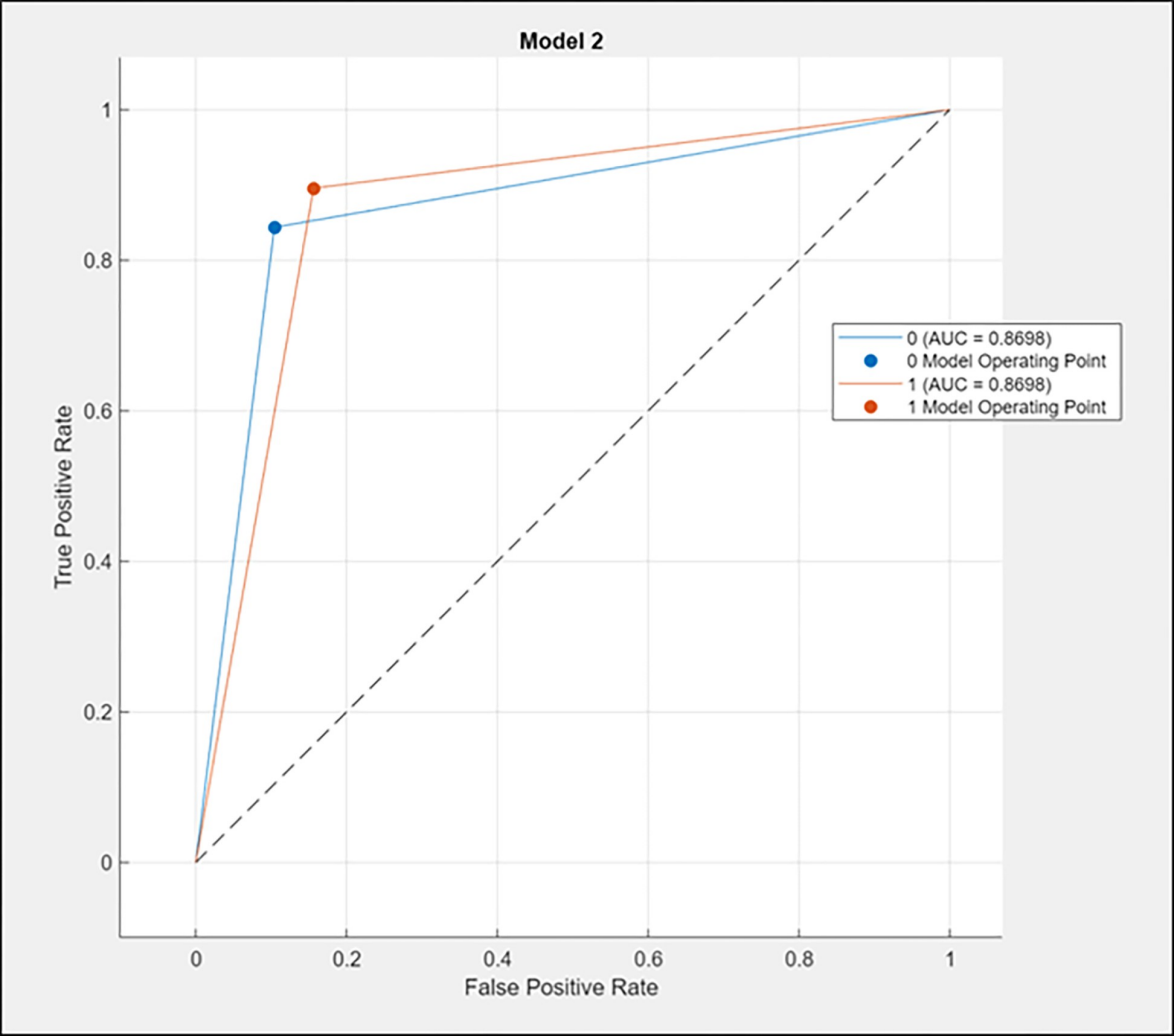
AUC-ROC curve.

AUC offers an overall performance metric across all potential classification criteria. The likelihood that the model values a random positive example higher than a random negative example is one approach to analyse AUC. The area under the ROC curve is 0.8698 for both classes of the tensile strength (0 for below the average value of the tensile strength, which is 744.13, and 1 for above the average value). The AUC-ROC curve shows the area under the ROC curve, as shown in Fig 4 below. Table 13 depicts the AUC-ROC values for all algorithms.

**Table 13:** AUC-ROC values.

Algorithm	AUC-ROC
kNN	0.8698
SVM	0.6204
Naïve Bayes	0.9375
Decision Tree	0.8454
Random Forest	0.7698
Logistic Regression	0.9303

## 4. Conclusion

This study mechanically evaluated several LPBF CM247LC samples with different process parameters through tensile testing. The study’s primary findings are as follows:

- The feature importance plot and heat map shows that volumetric energy density, laser power, and scan speed substantially impacted the tensile strength.
- The maximum value of as-built tensile strength obtained in the study is 997.81 MPa.
- Machine learning algorithms like k-nearest neighbours, Naïve Baiyes, Support vector machine, XGBoost, AdaBoost, Decision tree, Random forest, and logistic regression algorithms were 92.5%, 83.75%, 83%, 85%, 87.5%, 90%, 91.25%, and 77.5% accurate in classifying the tensile strength data.
- The current study obtained the best tensile strength at 235 W laser power, 850 mm/sec scan speed, and 0.11 mm hatch distance with 0.04 mm layer thickness, producing 62.83 J/mm^3^ volumetric energy density.

Future studies shall include how volumetric energy density influences the material’s microstructure, including grain size and orientation, affecting mechanical properties. The study will use hybrid machine learning models to develop the process-structure-property model for LPBF CM 247LC Alloy.

## References

[1] Sun Z. and Ion J.C., Laser Welding of Dissimilar Metal Combinations, Mater. Sci., 1995, 30, p 4205–4214.

[2] Kruth J.P., Leu M.C., and Nakagawa T., Progress in Additive Manufacturing and Rapid Prototyping, CIRP Ann., 1998, 47, p 525–540.

[3] Hilal H., Lancaster R., Jeffs S., Boswell J., Stapleton D., and Baxter G., The Influence of Process Parameters and Build Orientation on the Creep Behaviour of a Laser Powder Bed Fused Ni-based Superalloy for Aerospace Applications, Mater, 2019, 12, p 1390–1403. doi: 10.3390/ma12091390 31035638 PMC6539841

[4] Babu S.S., Raghavan N., Raplee J., Foster S.J., Frederick C., Haines M., et al. Additive Manufacturing of Nickel Superalloys: Opportunities for Innovation and Challenges Related to Qualification, Metall. Mater. Trans. A., 2018, 49, p 3764–3780.

[5] Markanday J.F.S., Applications of Alloy Design to Cracking Resistance of Additively Manufactured Ni-Based Alloys, Mater. Sci. Technol., 2022, 38, p 1300–1314.

[6] Wang X., Carter L.N., Adkins N.J.E., Essa K., and Attallah M.M., Novel Hybrid Manufacturing Process of CM247LC and Multi-Material Blisks, Micromachines, 2020, 11, p 492–509. doi: 10.3390/mi11050492 32408485 PMC7281307

[7] Santos E.C., Shiomi M., Osakada K., and Laoui T., Rapid Manufacturing of Metal Components by Laser Forming, Int. J. Mach. Tools Manuf., 2006, 46, p 1459–1468.

[8] Gu D.D., Meiners W., Wissenbach K., and Poprawe R., Laser Additive Manufacturing of Metallic Components: Materials, Processes and Mechanisms, Int. Mater. Rev., 2012, 57, p 133–164.

[9] Chapman N., Gray S., Sumner J., and Nicholls J., Stress Corrosion Testing of CMSX-4, CM247LC DS and IN6203DS Ni-Base Superalloys, Oxid. Met., 2021, 95, p 85–104.

[10] Gao Z., Zhang P., Niu Q., Li J., Nie L., Gong X., et al. Deformation mechanisms in the Directionally Solidified Nickel-Based CM247LC at Room Temperature, Philos. Mag. Lett., 2022, 102, p 190–199.

[11] Kumawat M.K., Parlikar C., Alam Md.Z., and Das D.K., Type-I Hot Corrosion of Ni-Base Superalloy CM247LC in the presence of Molten Na2SO4 Film, Metall. Mater. Trans. A., 2021, 52, p 378–393.

[12] Zhang P., Yuan Y., Gao Z.H., Li J., Niu Q., Shi X.B., et al. Microtwinning in the Nickel-Based Superalloy CM247LC during Compression Tests, Philos. Mag., 2022, 102, p 2235–2255.

[13] Wang D., Li S., Deng G., Liu Y., and Attallah M.M., A Melt Pool Temperature Model in Laser Powder Bed Fabricated CM247LC Ni Superalloy to Rationalise Crack Formation and Microstructural Inhomogeneities, Metall. Mater. Trans. A., 2021, 52, p 5221–5234.

[14] Meyer A., Daenicke E., Horke K., Moor M., Müller S., Langer I., et al. Metal Injection Moulding of Nickel-Based Superalloy CM247LC, Powder Metall., 2016, 59, p 51–56.

[15] Bidare P., Mehmeti A., Jiménez A., Li S., Garman C., Dimov S., et al. High-Density Direct Laser Deposition (DLD) of CM247LC Alloy: Microstructure, Porosity and Cracks, Int. J. Adv. Manuf. Technol., 2022, 120, p 8063–8074.

[16] Xu J., Gruber H., Lin Peng R., and Moverare J., A Novel γ′-Strengthened Nickel-Based Superalloy for Laser Powder Bed Fusion, Mater., 2020, 13, p 4930–4942. 10.3390/ma13214930 PMC7662829 33147831

[17] Rashid R., Masood S.H., Ruan D., Palanisamy S., Rahman Rashid R.A., Elambasseril J., et al. Effect of energy per layer on the anisotropy of selective laser melted AlSi12 aluminium alloy, Additive Manufacturing, Volume 22, 2018, Pages 426–439. doi: 10.1016/j.addma.2018.05.040

[18] Rashid R., Masood S.H., Ruan D., Palanisamy S., Rahman Rashid R.A., Brandt M., Effect of scan strategy on density and metallurgical properties of 17-4PH parts printed by Selective Laser Melting (SLM), Journal of Materials Processing Technology, Volume 249, 2017, Pages 502–511.

[19] Baturynska I., Semeniuta O., and Martinsen K., “Optimization of process parameters for powder bed fusion additive manufacturing by combination of machine learning and finite element method: a conceptual framework,” Procedia CIRP, vol. 67, pp. 227–232, 2018

[20] Marrey M., Malekipour E., El-Mounayri H., and Faierson E. J., “A framework for optimizing process parameters in powder bed fusion (pbf) process using artificial neural network (ann),” Procedia Manufacturing, vol. 34, pp. 505–515, 2019.

[21] Zhang X., Saniie J., and Heifetz A., “Detection of defects in additively manufactured stainless steel 316L with compact infrared camera and machine learning algorithms,” Journal of Occupational Medicine, vol. 72, no. 12, pp. 4244–4253, 2020.

[22] Gaikwad A., Giera B., Guss G. M., Forien J. B., Matthews M. J., and Rao P., “Heterogeneous sensing and scientifc machine learning for quality assurance in laser powder bed fusion–A single-track study,” Additive Manufacturing, vol. 36, Article ID 101659, 2020.

[23] Zouhri W., Dantan J. Y., Hafner B. et al., “Optical process ¨ monitoring for laser-powder bed fusion (L-PBF),” CIRP Journal of Manufacturing Science and Technology, vol. 31, pp. 607–617, 2020.

[24] Kwon O., Kim H. G., Kim W., Kim G. H., and Kim K., “A convolutional neural network for prediction of laser power using melt-pool images in laser powder bed fusion,” IEEE Access, vol. 8, pp. 23255–23263, 2020.

[25] Baumgartl H., Tomas J., Buettner R., and Merkel M., “A deep learning-based model for defect detection in laser-powder bed fusion using in-situ thermographic monitoring,” Progress in Additive Manufacturing, vol. 5, no. 3, pp. 277–285, 2020.

[26] Desai P. S. and Higgs C. F., “Spreading process maps for powder-bed additive manufacturing derived from physics model-based machine learning,” Metals, vol. 9, no. 11, p. 1176, 2019.

[27] Kou G., Xiao H., Cao M., and Lee L. H., “Optimal computing budget allocation for the vector evaluated genetic algorithm in multi-objective simulation optimization,” Automatica, vol. 129, Article ID 109599, 2021.

[28] Tijs L., Verhaeghe F., Craeghs T., Humbeeck J. V., and Kruth J. P., “A study of the microstructural evolution during selective laser melting of Ti–6Al–4V,” Acta Materialia, vol. 58, no. 9, pp. 3303–3312, 2010.

[29] ASTM E8/E8M-22, Standard Test Methods for Tension Testing of Metallic Materials. https://www.astm.org/e0008_e0008m-22.html

[30] Buhairi M.A., Foudzi F.M., Jamhari F.I. et al. Review on volumetric energy density: influence on morphology and mechanical properties of Ti6Al4V manufactured via laser powder bed fusion. Prog Addit Manuf 8, 265–283 (2023). doi: 10.1007/s40964-022-00328-0

[31] https://scikit-learn.org/stable/supervised_learning.html#supervised-learning

